# Putrescent Pulps: A Criticism

**Published:** 1902-11

**Authors:** 


### Domestic Correspondence.

### PUTRESCENT PULPS: A CRITICISM.

OCTOBER 3, 1902.

TO THE EDITOR:

SIR,—In your August issue there appears an article the caption of which is, "The Value of the Wenker Rubber Jaw as an Aid in teaching Therapeutics," in which is related an incident which is certainly amazing to read in a journal of that character, and more so as it was written by a *teacher* of operative dentistry in this the year of grace 1902.

The incident in question I will quote as follows: "I have a case in mind which will serve to illustrate. A graduate from a prominent school who has been practising for two years treated a putrescent incisor tooth for several months without any success. The putrescent condition was of short standing, and grew worse from the treatment. It became necessary to treat the tooth every day to keep the patient comfortable. If the dressing were sealed in the tooth the patient invariably suffered severely.

"The young dentist applied to an older practitioner for information as to the cause of the difficulty. Upon questioning, it was learned that he had been using antiseptics that were irritating and easily dissipated.

"The older practitioner advised him what to use, but after a short trial the young dentist gave up discouraged and brought the patient to the senior dentist for consultation. Upon removing the dressing the cotton was found so offensive that the odor remained in the office for a considerable time after the patient left. The canal was washed and rewashed with dioxygen until the mephitic gases were scarcely perceptible, and then dressed with eucalyptol and sealed.

"The young dentist returned in a few days and stated that the patient had been very comfortable since that treatment. The technic of the treatment was a revelation to him. He learned what to use, but more particularly *how* to use it. He confessed that he had a great deal of trouble with putrescent teeth, and believed that he would progress nicely after seeing that demonstration."

Here we have it related that a *recent graduate* of a *prominent school* consulted an *older practitioner* relative to the *protracted treatment* of a *putrescent pulp,* all of which is written up by a *teacher* in another (we trust, prominent) college, and all this is given hearing by such a body of practical (?) men as the Southern Wisconsin Dental Society, and last, but not least, occupies space in such a journal as the INTERNATIONAL.

Thus it is shown that a "prominent college" still believes in and teaches that protracted treatment is necessary for putrescent canals; that one of the recent graduates still pursues the same course, having only still more recently learned the process; that the "older practitioner" also consumes his own time and that of his patients by such a system of practice; that a professor in another college (the relator of the incident) also follows the same lines; that there is no evidence of any other practice being followed by any member of the association in question; and, lastly, that the INTERNATIONAL DENTAL JOURNAL gives such an "incident" the benefit of its pages without a protest.

As before stated, here is a string of almost incredible statements, and had the article in question been written by some backwoods dentist, say from the swamps of Louisiana, and appeared in a "border" journal, it would not have attracted my attention, but the circumstances are decidedly different.

Now, Mr. Editor, how can it be possible that these college professors, recent graduates, and older practitioners continue at this late day to *treat* putrescent root-canals, and if so, why? Do they not know that *immediate root-filling* in just such cases has been practised *successfully* for at least twenty years?

If I were advocating any original method, I surely would hesitate before writing in this way; but such is not the case.

So far as I am aware, Dr. Cassius M. Richmond is entitled to the credit of this method; at least, he "starred" through the country some twenty years or so ago, *demonstrating* its *success* upon every hand, and for this have I ever been most grateful.

Trusting that interest on the subject is sufficient excuse for the length of this letter, I remain,

Very cordially,

[REMARKS.—The foregoing letter came unsigned, but as the letter-head contains the name of Dr. C. Edmund Kells, Jr., New Orleans, it is presumed he is the writer.

The article which is so severely criticised, and also the INTERNATIONAL for admitting it without a protest, was published in the August number and read before the Southern Wisconsin Dental Society in May, 1902, by Dr. Harvey N. Jackson, of Milwaukee.

The INTERNATIONAL has a standing disclaimer on its first page that it is "not responsible for the views of authors," and were it to protest against everything that does not meet the views of the editor there would be but a limited time for other and more profitable work.

It is unquestionably true that the treatment of canals with putrescent pulps has not yet reached a stage when any one can dogmatize as to methods. Immediate root-filling is advocated by many, and finds equally strenuous opponents. The question is too large a one to be discussed here, but it may be said that any man who advocates the *immediate* root-canal filling in *putrescent* cases, as suggested by the critic, has not yet learned the *possibilities of septic poisoning*, and the occupant of the chair of pathology who would teach such a procedure certainly has missed his calling. While this is true, there can be no question but that canals in which putrefaction and consequently septic matter is not present had better be filled at once, and in no case where putrescence exists is it necessary for prolonged treatment.—ED.]

